# A Review of the Influence of Prebiotics, Probiotics, Synbiotics, and Postbiotics on the Human Gut Microbiome and Intestinal Integrity

**DOI:** 10.3390/jcm14113673

**Published:** 2025-05-23

**Authors:** Sylwia Smolinska, Florin-Dan Popescu, Magdalena Zemelka-Wiacek

**Affiliations:** 1 Department of Clinical Immunology, Faculty of Medicine, Wroclaw Medical University, 51-616 Wroclaw, Poland; magdalena.zemelka-wiacek@umw.edu.pl; 2 Department of Allergology, Nicolae Malaxa Clinical Hospital, Carol Davila University of Medicine and Pharmacy, 050474 Bucharest, Romania; florindanpopescu@gmail.com

**Keywords:** gut microbiome, microbiota, prebiotics, postbiotics, probiotics, synbiotics, epithelial barrier, intestinal integrity

## Abstract

Objective: This review aims to comprehensively evaluate the current evidence on the role of prebiotics, probiotics, synbiotics, and postbiotics—collectively referred to as “biotics”—in modulating the human gut microbiota and enhancing intestinal epithelial integrity. Findings: Biotics exert their beneficial effects through several mechanisms, including by promoting the growth of beneficial microbes, producing short-chain fatty acids (SCFAs), strengthening the gut barrier, and regulating immune responses. Prebiotics selectively stimulate beneficial bacteria, probiotics introduce live microorganisms with therapeutic functions, synbiotics combine the strengths of both, and postbiotics offer non-viable microbial components and metabolites that mimic probiotic benefits with enhanced safety profiles. Each type of biotic demonstrates unique and complementary effects across a range of conditions, such as inflammatory bowel disease, irritable bowel syndrome, obesity, constipation, and antibiotic-associated diarrhea. Implications: As disruptions in the gut microbiota and intestinal barrier are increasingly linked to chronic and immune-mediated diseases, leveraging biotics offers promising avenues for personalized nutrition, preventive healthcare, and adjunct therapies. The integration of biotics into clinical and dietary strategies may significantly contribute to improving gastrointestinal and systemic health.

## 1. Introduction

Microorganisms have been utilized in nutrition and medicine for millennia worldwide, well before humanity was aware of their existence. The gut microbiota plays an important role in regulating inflammatory, metabolic, immune, and neurobiological processes [1,2,3]. The potential of prebiotics, probiotics, synbiotics, and postbiotics in disease management is gradually being uncovered, revealing promising effects on the promotion of intestinal health, regulation of the gut microbiome, enhancement of epithelial integrity, and exploitation of immunoregulatory mechanisms. Understanding such complex effects and their mechanisms is essential for research strategies, which are still needed to further verify their long-term safety and efficacy, as well as to explore optimal therapeutic dosages and personalized treatment plans [3]. In addition to a scientific basis for therapeutic interventions, increased health awareness and consumer comprehension of the advantages of biotics has induced an increasing demand for such products. The market for probiotics, prebiotics, and postbiotics was valued at USD 57,074.2 million in 2023, and it is poised to expand at a compound annual growth rate of 7% from 2024 to 2030 [4].

The “biotics” family representatives that have been adequately identified and characterized as having a health benefit and being safe for their intended use are defined below with examples [4,5,6] (Table 1).

Prebiotics are substrates (devoid of live microbes) selectively utilized by healthy microorganisms resident in hosts to confer a health benefit [4,7,8,9,10,11]. Growth stimulation of *Lactobacillus* and *Bifidobacterium* spp. increases the production of short-chain fatty acids (SCFAs). Examples of prebiotics include the following fermentable oligosaccharide fibers: fructose-containing (fructans), such as inulin-type fructans (ITFs) and fructooligosaccharides (FOS), and galactose-containing, such as galactooligosaccharides (GOS) [9].

Probiotics are live microorganisms (of known origin, with a genome-sequencing confirmed identity and viability preserved through the end of shelf life) that benefit the host’s health when administered in efficacious defined adequate amounts [4,9,10,11,12]. Probiotic strains of non-spore-forming Gram-positive lactic-acid-producing bacteria (LAPB) inhabiting the gut of healthy adults and infants were originally isolated from fermented milk, as follows: genus *Lactobacillus* (phylum Firmicutes) and genus *Bifidobacterium* (phylum Actinomycetota/Actinobacteria). Examples include probiotic strains of LAPB, such as *Bifidobacterium animalis* subsp. *lactis* strain BB-12 and *Lacticaseibacillus*/*Lactobacillus rhamnosus* strain GG, as well as the probiotic non-pathogenic yeast *Saccharomyces boulardii* [9,12,13,14].

Synbiotics are a mixture of live microorganisms (probiotic or not necessarily proven probiotic) with a substrate(s) used for growth (prebiotic or not necessarily proven prebiotic) that are selectively utilized by resident microorganisms to benefit the host’s health [15]. They are classified as complementary synbiotics (mixture of proven probiotic + proven prebiotic) [4,9,16], for example, *Bifidobacterium animalis* subsp. *lactis* strain BB-12 + inulin and synergistic synbiotics (live microbes and substrate with synergistic effects) [17], such as *Lactobacillus rhamnosus* strain GG + tagatose.

Postbiotics are inanimate microbes and/or their components (intact non-viable microorganisms and/or microbial cell fragments/structures), with or without microbe-derived metabolites in the finished product [18]. Inanimate microbes are obtained by a controlled and reproducible deliberate processes of viability termination, as follows: heat (heat-killed), radiation, high pressure, or lysis. Examples include heat-killed and lyophilized *Lactobacillus acidophilus* human strain LB and fermented culture medium [19], heat-killed *Bifidobacterium bifidum* MIMBb75 [20], and pasteurized *Akkermansia muciniphila* [21].

This review was conducted as a narrative (non-systematic) literature review aiming to summarize and contextualize the current knowledge on the roles of prebiotics, probiotics, synbiotics, and postbiotics in human gut health and intestinal barrier integrity.

The human gut microbiota plays a vital role in health, and its modulation through biotics has been widely explored. Several reviews have summarized their individual benefits, particularly for metabolic and inflammatory conditions [1,11,22,23,24]. However, recent research highlights the importance of their combined effects, which remain less thoroughly assessed. This review updates the current understanding by integrating the findings on the mechanisms and clinical relevance of all four biotic types, addressing the gaps in the past literature, and highlighting the potential applications in disease prevention and gut barrier support.

## 2. Methods

Search Strategy: A comprehensive literature search was performed across the following electronic databases: PubMed, Scopus, Web of Science, and Google Scholar. The search covered studies published between January 2000 and 2024. Additional references were identified through citation tracking and manual searching of reference lists. The following keywords and Boolean operators were used in various combinations: “probiotics” OR “prebiotics” OR “synbiotics” OR “postbiotics” OR “biotics” AND “gut microbiome” OR “intestinal barrier” OR “epithelial integrity” OR “immune modulation” OR “dysbiosis” OR “short-chain fatty acids (SCFA)” OR “intestinal permeability” OR “gastrointestinal diseases”.

## 3. Pre-, Pro-, Syn-, and Postbiotics

### 3.1. Prebiotics

Prebiotics are non-digestible food components that beneficially affect the host by selectively stimulating the growth and/or activity of specific bacterial species in the gut (Table 2). They are typically composed of dietary fibers, such as fructooligosaccharides (FOS), galactooligosaccharides (GOS), and inulin [25]. The primary mechanism by which prebiotics exert their effects is through fermentation by gut microbiota, leading to the production of short-chain fatty acids (SCFAs), such as acetate, propionate, and butyrate [26]. SCFAs serve several vital functions. Butyrate, for instance, is a primary energy source for colonocytes and has anti-inflammatory properties. It enhances epithelial barrier function, thereby preventing pathogen translocation and maintaining intestinal integrity [27]. Moreover, SCFAs have systemic effects, including modulation of glucose and lipid metabolism and immune function [28,29].

Prebiotics significantly influence the composition and activity of the gut microbiome [54]. Prebiotics promote the proliferation of beneficial bacteria such as *Bifidobacterium* and *Lactobacillus*, which are known for their positive effects on gut health [55]. This shift in microbial composition can suppress the growth of harmful bacteria like *Clostridium perfringens* and *Escherichia coli*, thereby reducing the risk of gastrointestinal infections and inflammation.

### 3.2. Probiotics

Probiotics are live microorganisms that confer health benefits to the host when administered in adequate amounts (Table 3). Common probiotic strains include various species of *Lactobacillus*, *Bifidobacterium*, and *Saccharomyces* [10]. They can be obtained either in pure forms from various pharmaceutical companies or as key components of everyday foods, particularly fermented items, such as cheese, yogurt, and beer, among others [8]. These beneficial bacteria colonize the gut, outcompeting pathogenic microbes for nutrients and adhesion sites, thus inhibiting pathogen growth through competitive exclusion. Additionally, probiotics produce antimicrobial substances, such as bacteriocins and hydrogen peroxide, further suppressing harmful bacteria [56]. They also modulate the host’s immune response by enhancing the production of anti-inflammatory cytokines and promoting the activity of regulatory T cells, which help in maintaining immune homeostasis [56,57].

Probiotics and prebiotics, significantly influence the composition and activity of the gut microbiome [54]. Probiotics, on the other hand, introduce beneficial strains directly into the gut. These strains can enhance the diversity and stability of the gut microbiota, contributing to a balanced microbial ecosystem [80]. Probiotics can strengthen the gut barrier by increasing the production of mucin and upregulating tight junction proteins, which help prevent the translocation of pathogens and toxins into the bloodstream. Probiotics produce substances, such as lactic acid, hydrogen peroxide, and bacteriocins, which inhibit the growth of harmful bacteria. Through fermentation processes, probiotics produce SCFAs and other metabolites that influence gut health and the overall metabolism (reduce inflammation). Probiotics can also interact with the gut-associated lymphoid tissue (GALT) to modulate immune responses. They can enhance the production of anti-inflammatory cytokines and immunoglobulin A (IgA), which contribute to immune homeostasis [81]. This balance is crucial for preventing dysbiosis, a state characterized by microbial imbalance, which is associated with various diseases, including inflammatory bowel disease (IBD), irritable bowel syndrome (IBS), and metabolic disorders [82]. In diseased states, increased permeability allows pathogens to infiltrate, leading to inflammation and malabsorption.

To be considered a probiotic, a microorganism should have critical properties. It must be nonpathogenic, genetically safe, able to survive in the digestive system and produce beneficial secondary metabolites and immunomodulatory effects. Traditional probiotic microbes, such as *Bifidobacterium* spp. and *Lactobacillus* spp., are generally recognized as safe and are effectively used in many diseases, but they are not disease-specific. A new, crucial area of research is the characterization of more potent and specific next-generation probiotics (NGPs), such as *Akkermansia muciniphila*, *Bacteroides fragilis*, *Bacteroides thetaiotaomicron*, *Christensenella minuta*, *Clostridium butyricum*, *Eubacterium hallii*, *Faecalibacterium prausnitzii*, *Parabacteroides goldsteinii*, *Prevotella copri*, and *Roseburia intestinalis* [83].

### 3.3. Synbiotics

**Figure 1:**
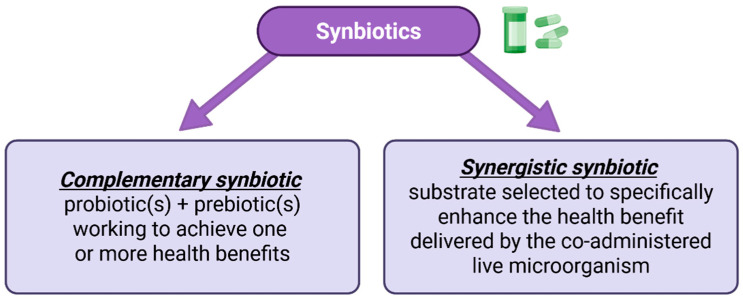
Categories of synbiotics.

In May 2019, the International Scientific Association for Probiotics and Prebiotics (ISAPP) convened a panel of nutritionists, physiologists, and microbiologists to review the definition and scope of synbiotics. The panel updated the definition of a synbiotic to “a mixture comprising live microorganisms and substrate(s) selectively utilized by host microorganisms that confers a health benefit on the host” [15]. Categories of synbiotics are described in Figure 1.

Synbiotics, preparations in which prebiotics are added to probiotics to achieve superior performance and benefits, have shown greater potential in improving intestinal function and enhancing host health compared to probiotics alone. There are still some issues in the clinical application of synbiotics, such as the individual differences, ratio of synbiotic components, and long-term safety assessments [3]. A particular approach to their formation is to induce prebiotic biosynthesis within the probiotic for synbiotic self-production or autologous synbiotics [84]. Future research is needed to explore the personalized application of synbiotics further, as well as their long-term effects and mechanisms of action [3].

Synbiotics, complex biotic preparations containing mixtures of live microbes and selected substrates, are divided into complementary synbiotics (consisting of probiotics and prebiotics with independent functions) and synergistic synbiotics (added microbes are specifically stimulated or their persistence or activity are enhanced by the cognate substrate prebiotics). Most commercial synbiotics, as well as those used in clinical trials, are of the complementary type, including Lactobacillus spp. and Bifidobacterium spp. as probiotics and FOSs, GOSs, and inulin-type fructans (IFTs) as prebiotics. Over the last years, synbiotics have been most commonly used in patients with metabolic disorders, including obesity, hypertension, and immune and gastrointestinal disorders. The complementary synbiotics applied to subjects with different gut environments reveal the need to apply different biotic products on an individual basis. Synergistic synbiotics may circumvent this issue, as such products combine bioactive components that can have beneficial effects even in non-responders to the complementary types [85,86].

### 3.4. Postbiotics

Postbiotics are a new type of biotic that hold great potential for improving health. According to the ISAPP, they are defined as a preparation of non-living/inanimate microorganisms and/or their components that confer a health benefit on the host [18] and may be administered at the host surface, e.g., oral cavity, nasopharynx, gut, skin, and urogenital tract.

Postbiotics may contain deliberately inactivated, inanimate, and intact microbial cells (progenitor microorganisms molecular characterized, with detailed description of inactivation procedure) and/or microbial cell components (cell fragments, structures, and sub-structures, such as plasma membrane lipids; cell wall compounds, including peptidoglycans and teichoic acids; extracellular biopolymers or exopolysaccharides; surface structures, such as flagella, pili, and fimbriae) with or without microbial metabolites/end-products, such as short-chain fatty acids (e.g., butyrate, acetate, and propionate), organic acids (e.g., indole-3-lactic acid), tryptophan metabolite, vitamins and antioxidative enzymes, and bacteriocins [23,87,88,89,90]. Unlike probiotics, postbiotics do not require living cells to induce health effects and are not subject to the food safety requirements that apply to live microorganisms. Cell-free supernatants or filtrates without cell components, purified microbial components (e.g., exopolysaccharides and peptides), or purified microbe-derived metabolites (e.g., organic acids), per se, are not considered postbiotics. Bioactive components such as exopolysaccharides and cell wall glycoproteins are, instead, considered parabiotics [18,91].

Though research into postbiotics is still in its early stages, there is growing evidence that they can improve gut health by strengthening the gut barrier, reducing inflammation, and promoting antimicrobial activity against gut pathogens.

## 4. Gut Health and Disease Management by Pre-, Pro-, Syn-, and Postbiotics

**Figure 2:**
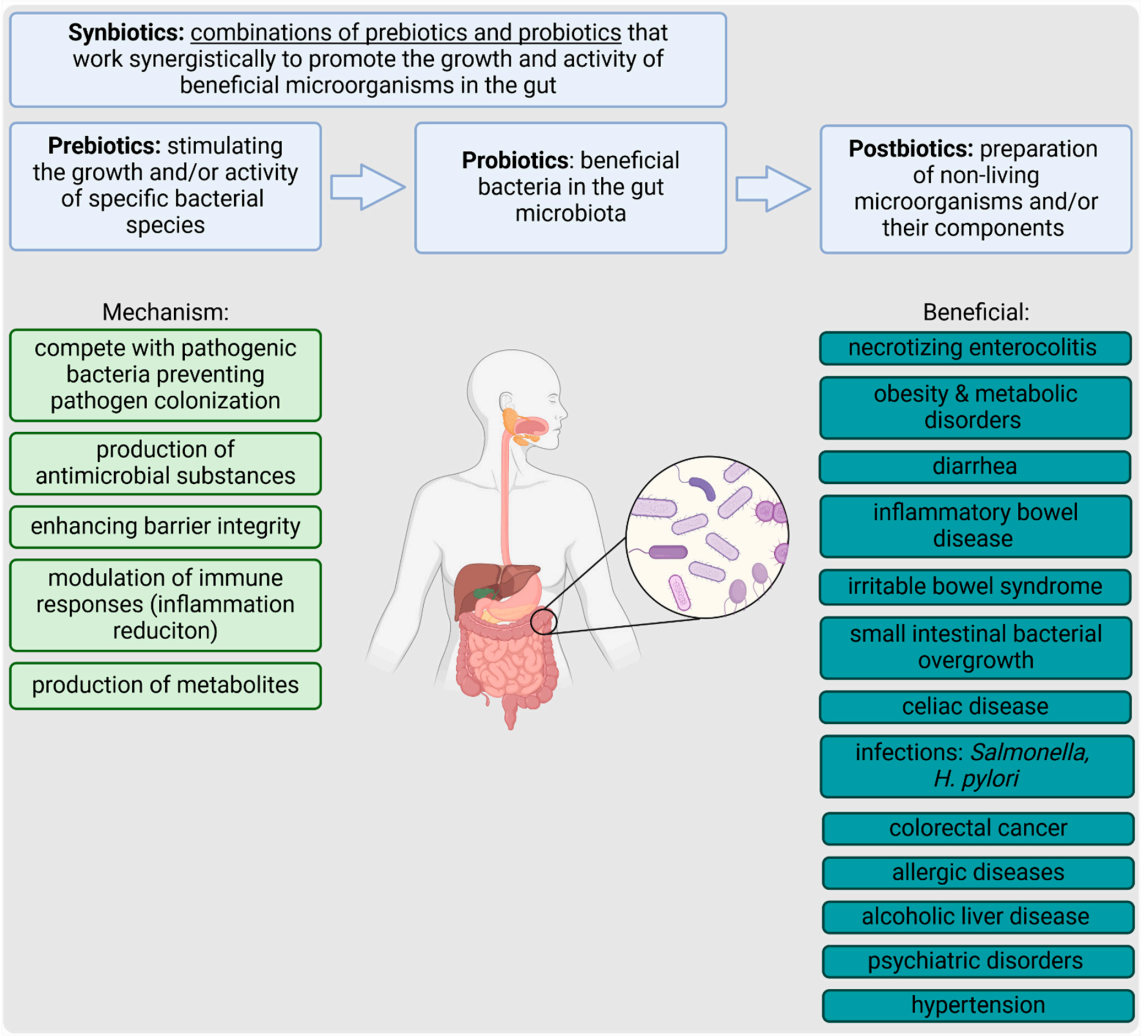
Presentation of the main roles of biotics in the human gut.

Biotics play crucial roles in maintaining gut health and managing diseases by modulating the gut microbiota, enhancing immune responses, and improving gut barrier functions (Figure 2).

Necrotizing enterocolitis (NEC), a major cause of illness and death in preterm infants, is not yet fully understood. However, gut dysbiosis, characterized by an imbalance between beneficial and harmful microbes, is believed to play a crucial role in its development. The therapeutic use of probiotics, prebiotics, and postbiotics shows promise in preventing NEC by modulating gut microbiota. Probiotics, especially *Lactobacillus* and *Bifidobacteria*, can enhance epithelial barrier function by upregulating tight junction proteins (tighten gut barrier), attenuate inflammation through nuclear factor kappa light-chain enhancer of activated B cells (NF-κB) pathway inhibition and support the growth of beneficial commensal bacteria [92,93]. These results support the beneficial effect of probiotics observed in meta-analyses of randomized and observational studies (real world data, RWD) [94].

Pro- and prebiotics have shown significant promise in managing obesity and related metabolic disorders through various mechanisms. Produced SCFAs stimulate the release of gut hormones, such as glucagon-like peptide-1 (GLP-1) and peptide YY (PYY), which enhance satiety and reduce food intake. Additionally, prebiotics influence systemic inflammation, a key factor in obesity, by reducing pro-inflammatory cytokines (e.g., tumor necrosis factor alpha (TNF-α) and IL-6), enhancing anti-inflammatory cytokines (IL-10), activating the G-protein coupled receptors (GPCRs), and regulates the gene expression of mucin family genes (MUC1–4) [95]. This immunomodulatory effect helps to decrease pro-inflammatory adipokines, which help regulate glucose and lipid metabolism, reduce systemic inflammation, and improve insulin sensitivity [96]. Prebiotics promote the growth of beneficial bacteria, further supporting this process by enhancing the integrity of the gut barrier, thereby reducing inflammation and endotoxemia (endotoxins are primarily derived from the cell walls of Gram-negative bacteria, which are commonly found in the gut microbiota) [97].

Probiotics have shown effectiveness in both preventing and treating various types of diarrhea through several mechanisms. In cases of infectious diarrhea, *Saccharomyces boulardii* CNCM I-745 has been found to significantly decrease the incidence of traveler’s diarrhea and reduce the duration of acute diarrhea [98,99]. This strain, along with others, such as *Limosilactobacillus reuteri* and *Bifidobacterium lactis*, competes with pathogenic microorganisms for adhesion sites on the intestinal mucosa, produces antimicrobial substances like bacteriocins, and modulates the host immune response to strengthen mucosal immunity [100,101]. For antibiotic-associated diarrhea (AAD), probiotics, such as *Saccharomyces boulardii*, *Lactocaseibacillus rhamnosus* GG, and multi-strain probiotic formulations, have been proven to significantly reduce the occurrence of AAD without increasing adverse effects [102,103]. These probiotics help restore the disrupted balance of gut microbiota caused by antibiotic therapy by promoting the growth of beneficial bacteria, enhancing gut barrier function, and modulating inflammatory responses through the production of SCFAs and other metabolites [104,105]. For diarrhea induced by chemotherapy and radiation therapy, probiotics, such as *Lactobacillus acidophilus*, *Bifidobacterium bifidum,* and *Saccharomyces* species, have been shown to reduce both the incidence and severity of diarrhea by maintaining gut microbiota diversity, enhancing mucosal barrier function, and modulating immune responses to decrease intestinal inflammation and damage [106,107]. These probiotics increase the production of SCFAs, which lower gut pH, inhibit pathogen growth, and promote the healing of the intestinal mucosa by stimulating mucus production and tightening epithelial junctions [108]. Also, prebiotics, like FOS, were found to effectively modulate gut microbiota composition and metabolism in children with functional diarrhea, increasing beneficial bacteria and inhibiting harmful bacteria, thus providing a potential treatment to alleviate symptoms and reduce antibiotic use [109].

Prebiotics and postbiotics have shown promise in the management of inflammatory bowel disease (IBD) due to their ability to modulate the gut microbiota and reduce inflammation. Prebiotics, such as inulin and FOS, serve as substrates for beneficial gut bacteria, promoting the production of SCFAs, which are known to enhance intestinal barrier function, block epithelial binding, or inhibit colonization of pathogenic bacteria. Pre- and probiotics exert anti-inflammatory effects by inducing regulatory T cells and inhibiting pro-inflammatory pathways—like NF-κB activation in macrophages and oxidative stress or production of TNF-α, decrease luminal pH and enhance the production of IL-10, TGF-β, and IgA [110,111]. Prebiotics, particularly FOSs and germinated barley foodstuffs, demonstrate potential as effective and safe dietary supplements for inducing and maintaining remission of IBD [112,113]. Pre- and probiotics offer similar therapeutic potential for irritable bowel syndrome (IBS) by modulating the gut microbiota and enhancing gut–brain communication, as well as alleviating IBS symptoms, including abdominal pain and bloating, through mechanisms like immunomodulation and improved mucosal integrity [114,115,116,117].

Synbiotic administration to IBD patients results in beneficial therapeutic effects. Prebiotic synergy 1, in combination with *Bifidobacterium longum*, improved sigmoidoscopy scores and reduced b-defensins, TNF-α, and IL-1α in biopsy samples from ulcerative colitis patients. Patients who received *Bifidobacterium longum* and prebiotic synergy 1 (with FOS/inulin blend) combination revealed a significant histological improvement in comparison to the placebo group. Synbiotics significantly reduced TNF-α expression, thereby indicating their potential beneficial effect in the management of IBD. Combinations of synbiotics may exert beneficial impacts on the intestinal mucosa [111].

In patients with constipation, alterations in the gut microbiome are often observed, including decreased levels of *Bifidobacteria* and *Lactobacilli* and increased levels of *Bacteroides*. The therapeutic potential of prebiotics and probiotics in managing constipation is linked to their ability to modulate these microbial populations [118]. Prebiotics, such as inulin and GOSs, enhance the growth of beneficial bacteria, leading to increased production of SCFAs, which stimulate colonic motility by promoting serotonin release and activating enteric or vagal nerves, thereby improving bowel movements [119,120]. Clinical studies have shown that specific prebiotics, like chicory inulin and Deshipu stachyose granules (DSGs), significantly increase stool frequency and improve stool consistency and ease of defecation in constipated individuals [121]. Probiotics, particularly strains like *Bifidobacterium lactis* and *Lactobacillus acidophilus* and *sakei*, have also demonstrated efficacy in alleviating constipation [122,123]. These probiotics enhance stool frequency and consistency by modulating gut motility and improving gut barrier function [124]. For example, supplementation with *Streptococcus thermophilus* and *Lactiplantibacillus plantarum* has been shown to significantly enhance stool consistency and maintain these benefits post-treatment [125]. Multi-strain probiotic formulations tend to be more effective than single-strain preparations, improving defecation frequency and reducing symptoms of constipation [126,127].

In small intestinal bacterial overgrowth (SIBO), antibiotics are commonly used for treatment, but frequent recurrences necessitate repeated treatments, increasing the risks of antibiotic resistance, diarrhea, and food intolerances. Probiotics are being explored as treatment due to their ability to produce antimicrobial substances, compete with pathogens, enhance gut motility, and restore gut microbiota balance post-antibiotic therapy [128,129].

While the primary treatment for celiac disease is a lifelong gluten-free diet, this alone often does not fully restore gut microbiota. Probiotics, particularly strains like *Bifidobacterium* and *Lactobacillus*, have shown potential in helping restore gut microbiota balance, pre-digesting gluten in the intestinal lumen, reducing inflammation, improving intestinal permeability, and modulating cytokine and antibody production. These effects can enhance symptoms and quality of life in celiac disease patients [130,131].

In *Salmonella* infection, strains such as *Lactobacillus* and *Bifidobacterium,* in particular, can compete with *Salmonella* for nutrients and adhesion sites on the gut lining, thus preventing the pathogen from establishing an infection [132,133]. Similarly, in *Helicobacter pylori* infection, probiotics like *Lactobacillus rhamnosus* have been shown to inhibit *H. pylori* by producing lactic acid and other antimicrobial compounds, enhancing mucosal immunity, and reducing inflammation. Pre- and postbiotics can support the growth of these beneficial microbes, further helping to maintain a balanced gut microbiota and can be helpful in eradication [134,135,136].

Excessive use of antibiotics has led to the recent rise in multidrug antibiotic resistance, often accompanied by gut dysbiosis. To avoid such harmful effects, probiotics have emerged as an effective intervention. Prebiotics and postbiotics, including parabiotics, have also gained significant attention. Prebiotic dietary fibers are selectively fermented by probiotics, promoting their proliferation in the gut. Postbiotics containing fermentation products and parabiotics, such as exopolysaccharides and cell wall glycoproteins, impact the growth of harmful pathogens by lowering the pH, producing bacteriocins, and inhibiting the adhesion and biofilm formation of pathogens on the intestinal epithelium [91].

Additionally, prebiotics, probiotics, and synbiotics have shown potential in adjuvant cancer therapy, particularly colorectal cancer, by maintaining the colon barrier, regulating the immune system, and counteracting the toxic side effects of chemotherapy [137,138].

Probiotics may also be valuable in treating alcoholic liver disease due to their ability to enhance liver function, decrease inflammation, and regulate gut flora [3,139]. The therapeutic potential of probiotics, particularly next-generation probiotics, in non-alcoholic fatty liver disease/metabolic dysfunction-associated steatotic liver disease has also been recently discussed [140].

*Lactobacillus* species revealed several effects on immunological parameters in allergic diseases. Their modulatory effects seem strain-dependent. However, the exact mechanism still needs to be elucidated, and no specific *Lactobacillus* strain emerged as the most efficient one. Since some human studies have found significant effects of *Lactobacillus* strains on allergic rhinitis symptoms, and no adverse effects have occurred, the probiotic bacteria treatment seems suitable for allergic rhinitis patients [141]. Some *Lactobacillus* strains can serve as effective immunobiotics in allergic rhinitis by maintaining the T-helper lymphocytes (Th)1/Th2 balance via modulating the functions of various cytokines and chemokines [142]. Moreover, probiotic intervention with a *Lactobacillus* strain mixture increases the expression of the high-mobility group nucleosome-binding domain-containing member of the high-mobility group nucleosome-binding domain (HMGN) family of proteins, also known as the non-histone chromosomal protein HMG-17 [143]. A systematic review revealed that probiotics improve quality of life and symptom scores in allergic rhinitis [144].

Food allergy develops from a defect in immune tolerance mechanisms. These are modulated by the gut microbiome composition and function and intestinal dysbiosis has been associated with the development of food allergy. Selected probiotic strains could regulate immune tolerance mechanisms, but these are multiple and are still not thoroughly clarified. Increased evidence is needed to provide practical information on the choice of optimal bacterial species/strains, dosage, and timing for intervention [145].

Next-generation probiotics like *Akkermansia muciniphila* and synbiotics may support the management of psychiatric and hypertensive disorders via microbiota-related pathways, but current evidence on their efficacy and safety remains limited [146,147]. Growing interest in postbiotics derives also from the fact that dysbiosis and disruption of the intestinal barrier function are linked to various conditions, including inflammatory bowel disease, irritable bowel syndrome (IBS), obesity, celiac disease, and food allergies [148].

Postbiotics’ applications in human health include alleviating diarrhea in children and reducing *Helicobacter pylori*, targeting manifestations of irritable bowel syndrome, constipation, chronic diarrhea, bacterial vaginitis, and obesity in adults [149]. Further research will determine the exact potential of postbiotics in treating inflammatory bowel disease, including ulcerative colitis, Crohn’s disease, and celiac disease [150,151]. The role of postbiotics as innovative strategies for the prevention and treatment of food allergy has also begun to draw the great attention of scientists [152].

Postbiotics are introduced into the pharmaceutical, veterinary, and food industries (as medication, food, and feed) for the prevention and treatment of specific diseases, boosting animal health status, and producing functional foods. Potential applications of postbiotics in animals, including ruminants and monogastric animals, are related to improving gut barrier function and microbiota modulation, for enhancing growth, gut health, and overall productivity. Postbiotics are of particular interest in the pharmaceutical industry and also in the food industry, where they can help preserve and improve the nutritional properties of food [149,153].

A summary of the representative clinical studies evaluating the effects of biotics on gut barrier function is presented in Table 4.

## 5. Biotics and Intestinal Epithelial Integrity

Prebiotic GOSs and FOSs have protective effects on epithelial damage in heat/hypoxia-exposed human Caco-2/HT-29 colonic cells (derived from a human colorectal adenocarcinoma) by preventing the decrease in trans-epithelial electrical resistance, the increase in paracellular permeability, and/or decrease in TJ proteins zonula occludens-1 (ZO-1) and claudin-3 expression [158]. FOS prebiotics, known to regulate intestinal barrier function, stimulate tight junction assembly in human T84 intestinal epithelial cells via a calcium/calmodulin-dependent protein kinase β-AMP-activated protein kinase (CaMKKβ-AMPK) pathway [159]. In addition, FOS reverse the ability of lipopolysaccharide to suppress AMPK activity and tight junction assembly. This effect of FOS offers an explanation for the positive impact observed in experimental models of inflammatory intestinal diseases [160]. It also lays the groundwork for the development of FOS as a potential therapy for diseases characterized by tight junction disruption in intestinal epithelia. Moreover, inulin-type fructans (ITFs) prevent the T84 intestinal epithelial barrier disruption induced by calcium ionophore A23187 and decrease the production of IL-8 induced by the mentioned barrier disruptor [161]. In addition, prebiotics such as FOS differentially shift microbiota composition and function and improve intestinal epithelial barrier in vitro [162]. The prebiotic longish glucomannan hydrolysates (LGHs), developed to improve the intestinal mucosal barrier, also induce local protective immunity with CD3^+^ T cells infiltrating the epithelium for cell repairs and CD4^+^ T cells agglomerating in the isolated lymphoid follicles for immune modulation, these responses being probably coordinated with innate lymphoid cells type 3 (ILC3) participation [163].

In a sucralfate-induced constipation mouse model, the probiotic *Lacticasei bacillus rhamnosus* M15 was recently shown to recuperate the colonic epithelial integrity [164], similarly to *Lactobacillus plantarum* NCU116, which was previously revealed to increase restoration of colonic mucosa in a loperamide-induced constipation in mice [165]. *Clostridium butyricum*, in combination with germinated barley fibers, also suppressed crypt loss and inflammatory processes in dextran sulfate sodium-induced experimental colitis in rats, this being associated with its high activity in increasing SCFA levels in the gut lumen [166]. SCFAs are considered to be essential for the integrity of the colonic epithelium by stimulating the proliferation of the epithelial colonic cells and by being a major source of energy for the enterocytes, particularly butyrate [167]. Therefore, it appears that probiotics decreased mucosal damages induced partly due to the marked production of SCFAs [166]. A systematic review of randomized controlled trials and animal studies in overweight and obese individuals revealed that probiotics, such as *Bifidobacterium*, *Lactobacillus*, and *Akkermansia*, effectively reduce intestinal permeability and improve gut barrier function. Nevertheless, better standardization of strain use, dosage, duration, and delivery matrix is needed to understand the probiotic impact on intestinal permeability thoroughly [168].

The synbiotic effects of dietary fibers and lactobacilli, such as long-chain inulin and Lactobacillus acidophilus W37, are not limited to the effects on gut microbiota but can also occur by synergistically directly stimulating intestinal epithelial cells, [163]. In a murine model of inflammatory bowel disease with a synbiotic treatment consisting of probiotic *Bacillus coagulans* MTCC5856 spores and prebiotic whole plant sugar cane fiber, an immunohistochemical analysis was performed to evaluate the assembly of the tight junctions (TJs) and the integrity of the intestinal barrier. Basolateral and partial apical staining of TJ proteins zonula occludens-1 (ZO-1), occludin, and claudin-1 was maintained with the probiotic *B. coagulans*, while prebiotic fibers were able to partially maintain ZO-1 and claudin-1 staining, such an effect being less evident for occludin. The synbiotic treatment was most effective in preserving the TJ protein expressions, confirming beneficial effects on the intestinal integrity in a dextran sulfate sodium-induced colitis mice model [169]. In a rat constipation model experiment in which colonic epithelial integrity was analyzed by a digital image analysis system revealed that a synbiotic treatment consisting of a combination of *Bifidobacterium lactis* BB12, *Lactobacillus plantarum* LP01, and inulin-oligofructose inhibited local inflammatory responses and recovered the colonic epithelial integrity (repristinating the colonic wall and villi integrity) [170].

Postbiotics significantly shape the host intestinal microbiota by creating a more favorable and protective microbial community composition. Their beneficial effects are elicited via several potential mechanisms involving bacteriocins, organic acids, and short-chain fatty acids (SCFAs), pili and fimbriae, direct bacterial coaggregation, or structural disruption induced by non-viable *Lactobacillus* spp. [149]. A remarkable characteristic of postbiotics is their ability to have antimicrobial activity despite being derived from inanimate bacteria. Bacteria with the ability to produce bacteriocins and other antimicrobial molecules may be integrated into postbiotics in the form of cell lysates or metabolites [171]. Some postbiotics contain bacteriocins as ribosomally synthesized antimicrobial peptides that can inhibit the growth of microbial pathogens [172]. Bacteriocins are active against other bacteria, either belonging to the same species (narrow spectrum) or even across genera (broad spectrum). Producing microorganisms are resistant to their bacteriocin(s), characteristic mediated by specific proteins [173]. As bacteriocins have a bactericidal mode of action, usually targeting the cytoplasmic membrane, there is no cross-resistance with antibiotics [174]. The latest and updated classification system of bacteriocins suggests two large classes. Class I includes post-translationally modified peptides (RiPPs), such as nisin (lantibiotic) from *Lactococcus lactis,* targeting *Staphylococcus aureus* and *Clostridium difficile*; gassericin A (circular peptide) from *Lactobacillus gasseri* targeting *Listeria monocytogenes*, *Bacillus cereus,* and *Staphylococcus aureus*; and microcin C (nucleotide peptide) from *Escherichia coli* Nissle 1917, targeting *Escherichia coli* O157 and *Salmonella* typhimurium. Class II bacteriocins are unmodified bacteriocins, such as pediocin PA-1 from Pediococcus acidilactici against *Listeria monocytogenes* and plantaricin MG from *Lactiplantibacillus plantarum* subsp. *plantarum* targeting *Listeria monocytogenes* and *Salmonella typhimurium*. Conventionally, class I and II bacteriocins are pH and heat stable and, thus, can still perform their antimicrobial function after exposure to heat [175,176,177]. Antimicrobial components of postbiotics, obtained from bacteriocin-producing bacteria, have the potential to effectively inhibit notable pathogens, such as *Bacillus cereus*, *Enterococcus faecalis*, *Listeria monocytogenes*, *Streptococcus faecalis*, *Staphylococcus aureus*, *Salmonella typhimurium*, and *Escherichia coli* [149].

SCFAs are a subset of fatty acids that are produced by the gut microbiota during the fermentation of partially and non-digestible polysaccharides. Moreover, some postbiotics indirectly modulate the gut environment by introducing organic acids, including lactic acid, along with SCFAs, such as acetic, propionic and butyric acids, which by lowering pH levels within the digestive tract enhance the proliferation of beneficial microorganisms such as lactic acid bacteria (LAB) and *Bifidobacteria* while concurrently impeding the proliferation of pathogens like *Enterobacteria*, *Escherichia coli*, and *Salmonella* [178,179]. In addition, the exopolysaccharide (EPS) of *Levilactobacillus brevis* M-10 lowers intestinal pH and can be utilized by gut microbes to produce SCFAs, such as butyric acid and propionic acid [180]. Moreover, postbiotics may incorporate elongated filamentous protein structures that enhance adhesion to particular locations, thus facilitating the establishment of beneficial microbial populations. Pili and fimbriae are such structures that protrude from the bacterial cell walls of both Gram-negative and Gram-positive bacteria. The multisubunit pili SpaCBA of *Lactobacillus rhamnosus* GG is a key factor involved in adherence to human intestinal epithelial cells, biofilm formation, and diminishing of proinflammatory cytokine IL-8 mRNA expression in epithelial cells provoked by other cell surface components, such as lipoteichoic acid (LTA) via TLR2 interaction [181]. Other postbiotics have the ability to coaggregate with microbial pathogens or disrupt their structure and integrity [149]. Non-viable *Lactobacillus reuteri* DSMZ 17648 in adult humans revealed reduced *Helicobacter pylori* load without adverse effects. This particular strain co-aggregates with the flagellated helical bacterium without interfering with other bacteria of the commensal intestinal flora. Such a specific binding may mask the surface structures of *Helicobacter pylori* and interfere with its motility. The aggregated pathogens assumably no longer adhere to the gastric mucosa, thus being cleared from the stomach [182]. As an additional mode of action, *Lactobacillus reuteri*, which shares glycolipid-binding specificity with *Helicobacter pylori*, might be an effective competitor to pathogens at the molecular receptor level [183]. The non-viable heat-killed strain HK-LJ88 of *Lactobacillus johnsonii* No.1088 induces structural changes with deformations of *Helicobacter pylori*, including bending of the cell body, disappearance of the spiral, degradations, and coccoid formation, not associated with coaggregation phenomenon [184].

The positive impact of postbiotics on gut microbiota composition consists in promoting the growth of beneficial species while suppressing pathogenic bacteria, thus contributing to the maintenance of a healthy balanced intestinal environment. The increase in lactic acid bacteria populations facilitated by postbiotics also contributes to the competition between beneficial and pathogenic bacteria in the gut environment, leading to a decline in the number of pathogens [181]. Postbiotics modulate mucus-associated microbiota, with inhibition of *Clostridia* in the ascending colon and proliferation of *lactobacilli* in the descending colon. In addition, postbiotic module luminal microbiota with inhibition of *Coliforms*, *Clostridia*, *Staphylococci*, and facultative anaerobes in the ascending and transverse colon, and growth stimulation of *Enterococci* in the transverse and descending colon [149]. Finally, it should be noted that individuals who received postbiotics had a higher abundance of beneficial microbes and a lower abundance of pro-inflammatory bacteria, as revealed by recent fecal metagenomics analysis. These data are of interest to food scientists, clinicians, and the health food industry [185].

The gut barrier is one of the most important barriers between the host and the external environment (including diet, drugs, pathogens, and microbiota) [177]. Maintaining intestinal epithelial barrier (IEB) integrity is essential for human health. It protects against invading allergens, toxins, and pathogens while preserving the fragile balance between commensal microorganisms and the immune system, which helps maintain homeostasis and is crucial for overall health [149]. To study postbiotics’ effects on intestinal epithelial cells, an in vivo experimental model with IL-10-deficient mice using two lactic acid bacterial strains, *Bifidobacterium breve* C50 and *Streptococcus thermophilus* 065, the treatment with bacteria-conditioned medium had a positive effect on the epithelial barrier, as revealed by a reinforcement of the distal colonic barrier, both at the transcellular and paracellular levels [186]. Postbiotics derived from *Lactobacillus paracasei* enhance the mucin-2 (MUC2) expression in murine models of constipation, thus supporting the maintenance and repairing of IEB. This mucin secreted by goblet cells and glands throughout the gastrointestinal tract has a crucial role in maintaining the gut barrier function [187,188]. Postbiotics may strengthen the epithelial barrier by several mechanisms, such as the enhancement of tight-junction functioning, induction of mucin secretion, and prevention of apoptosis of epithelial cells [189]. In order to modulate the epithelial cell function by increasing tight junction integrity, there are important interactions with the metabolites and other bioactive molecules in postbiotics [190]. Various host reactions to postbiotic components, such as exopolysaccharide (EPS) fraction, SCFAs, LTA, secreted proteins, surface layer proteins, and bacteriocins, are interconnected, working collectively to maintain intestinal epithelial homeostasis in complex environments. Postbiotics play a protective role in maintaining IEB function similar to probiotics, [149] and represent a valid alternative to probiotic strains for preserving a healthy IEB [191]. Introducing postbiotic products to replace live probiotics was suggested to avoid the potential risks in certain conditions. For special individuals, such as immunocompromised patients, preterm infants and those with low birth weight, administration of probiotics must be conducted very carefully, because some probiotics are reported to induce bacteremia and sepsis [192,193].

Exopolysaccharide EPS116 from Lactobacillus plantarum NCU116 promotes epithelial barrier function and the expression of tight junction (TJ) proteins in vitro and in vivo, by the upregulation of key proteins ZO-1 and occludin, while repressing the expression of the tight junction protein claudin-2 and pro-inflammatory cytokines, including IFN-γ, IL-6 and TNF-α. The regulation of epithelial barrier function by EPS116 is STAT3 dependent [194]. B-EPS, the exopolysaccharide-enriched fraction from Bacillus subtilis J92, also restores the intestinal barrier integrity by modulating tight junction-related proteins, such as occludin, claudin-1 and claudin-2, and epithelial–mesenchymal transition marker proteins including E-cadherin and N-cadherin. Furthermore, B-EPS downregulates inflammatory cytokines, such as IL-6 and IL-1β, by involving the transcription factors NF-κB and STAT3 [195]. Similarly, purified EPS produced by Streptococcus thermophilus MN-BM-A01, composed of rhamnose, glucose, galactose, and mannose, improves the mucosal barrier function by enhancing the expression of tight junction proteins claudin-1, occludin, and E-canherin, while repressing pro-inflammatory cytokines such as IL-6 and IFN-γ [196].

Short-chain fatty acids (SCFAs), which are free fatty acids (FFAs) with fewer than six carbon atoms in their aliphatic structure, are the major microbial metabolites from the bacterial fermentation of dietary fibers produced in the intestine. SCFAs are a subset of fatty acids that represent end-products of the anaerobic fermentation of partially and non-digestible polysaccharides by intestinal commensal microbiota and the major energy source for intestinal epithelial cells. The main SCFAs produced in the gut are acetic acid (C2), propionic acid (C3), and butyric acid (C4), and they represent 95% of all SCFAs in mammals. [197] SCFAs’ highest levels are found in the proximal colon, where they are used locally as energy source by enterocytes or transported across the intestinal epithelium into the bloodstream. The predominant SCFAs present at high levels in the colon (butyrate), entero-hepatic circulation (propionate), and systemic circulation (acetate) are responsible for epithelial protection and regulation of the inflammatory intestinal responses [198]. SCFAs can modulate epithelial cell functions either by inhibition of histone deacetylases (HDACs) activities (mainly butyrate) with induction of the transcription of specific genes supporting intestinal epithelial homeostasis and apoptosis, or by activation of ‘metabolite-sensing’ G-protein-coupled receptors (GPCRs) [199,200,201]. SCFAs effects are mediated by such *free fatty acid receptors*, as follows: GPR43 (FFAR2), GPR41 (FFAR3), and GPR109A (hydroxycarboxylic acid receptor HCAR2) expressed on immune cells and a variety of tissues including intestinal epithelial cells [202,203]. GPR43 is activated by the three main SCFAs (acetate, propionate, and butyrate) with similar affinities. It activates the phospholipase-Cβ, which releases intracellular calcium and stimulates protein kinase C in addition to cAMP accumulation inhibition and protein kinase A and ERK activation. GPR109a activation by butyrate and GPR41 activation by propionate and butyrate induces the inhibition of cyclic adenosine monophosphate (cAMP) accumulation and protein kinase A and mitogen-activated protein kinases (ERK and p38) activation [204]. SCFAs contribute to IECs integrity through the upregulation of tight junction proteins, stabilization of HIF transcription factor, and NLR pyrin domain 3 (NLRP3) inflammasome modulation [199]. SCFAs, alone or in combination, significantly increase transepithelial electrical resistance (TER) and stimulate the formation of tight junctions. Bacteria-derived butyrate enhances IEB function via increasing the tight junction claudin-1 transcription by facilitating the interaction between transcription factor SP1 and a specific motif within the promoter region of claudin-1 [205]. SCFAs protect the IEB from disrupting lipopolysaccharide (LPS) via inhibiting NLRP3 inflammasome and autophagy. Moreover, SCFAs act as HDAC inhibitors to suppress NLRP3 inflammasome and act as energy substances to protect IEB and inhibit autophagy [206]. SCFAs affect epithelial O_2_ consumption resulting in stabilization of hypoxia-inducible factor (HIF), a transcription factor coordinating IEB protection [207]. In addition, SCFAs ensure a low antibacterial pH around epithelial cells and favor mucus synthesis [199].

Lipoteichoic acid (LTA) is a vital surface component of the cell wall of lactobacilli, involved in key cellular and immunomodulatory functions. In LPS-stimulated human colonic HT-29 cells with epithelial morphology, the LTA induced a noticeable increase in IL-10 and reduced TNF-α levels. In a colitis mouse model, LTA as a postbiotic component derived from *Lactobacillus* strains reduced gut permeability. This effect may be due to the interaction between LTA and toll-like receptor TLR-2, leading to the upregulation of tight junction (TJ) proteins in the epithelium and the expression of the zonula occludens ZO-1 gene [149,208].

Important secreted cell wall proteins are functional muramidases present in *probiotic Lactobacillus* spp., such as pp75 (75 kilodaltons) and p40 (40 kilodaltons) [209]. These bacterial soluble proteins produced by *Lactobacillus rhamnosus GG* (LGG) regulate the homeostasis of the intestinal epithelial cells through specific cellular signaling pathways, involving Akt and p38 MAPK, and help to restore intestinal epithelial integrity through not only preventing apoptosis but also enhancing proliferation. Such postbiotics-purified proteins were tested on murine colon organ explants and placed on netwell culture filters, with or without TNF-α as inflammatory stimuli. In such an experimental ex vivo mouse model, p75 and p40 help restore colonic epithelial integrity after TNF-induced injury and the colonic crypt structures in cultures stimulated with TNF-α [210].

Ileal and colonic human explants have been used to study the potential benefits of *Lactobacillus* postbiotics [211]. Many studies in mouse models have reported the protective effects of *Lactobacillus rhamnosus* GG culture supernatant against gut barrier injury caused by chemicals, such as alcohol, dextran sodium sulfate, and hydrogen peroxide [212,213,214,215]. Moreover, pre-treatment with *Lactobacillus rhamnosus* GG postbiotics abrogates the deleterious effects of *Escherichia coli* K1 on intestinal integrity in neonatal rats. Such postbiotics have considerable potential in promoting the maturation of neonatal intestinal defense, including the upregulation of the Ki67 marker or proliferative cells, as well as goblet-cell-produced mucin MUC2 and immunoglobulin IgA [216]. The postbiotic derived from *Lactobacillus plantarum* RG14 has a high antioxidant activity corelated to greater glutathione peroxidase (GPX) in lambs serum [217]. Although dissimilar to p40 and p75, HM0539 has a distinct role in intestinal barrier protection. This postbiotic from the culture supernatant of LGG was also found to protect intestinal epithelium from LPS- or TNF-α-induced injury [218,219]. HM0539 exhibits a potent protective effect on the intestinal barrier by downregulation of intestinal MUC2 and ZO-1, as well as disruption of the intestinal integrity [220]. Some researchers consider p40 and p75 to be the most abundant proteins purified from LGG culture supernatant [210].

Although both p40 and p75 have the potential to modulate intestinal homeostasis, p40 exerts more potent effects than p75 [221]. p40 can inhibit cytokine-induced intestinal epithelial apoptosis, enhances intestinal mucin and IgA production, thus preserving IEB function [222,223,224]. Different researchers found p75 as the most abundant protein [225], while others identified at least 58 proteins from LGG supernatant, among which HM0539 was the most abundant [220]. These varying results may be due to the different procedures in preparing LGG culture supernatant, because culture conditions may affect the secreted microbial proteins [226].

The surface layer protein S-layer from the heat-inactivated strain *Lactobacillus helveticus* ATCC 15009 as postbiotic improves transepithelial electrical resistance (TEER) and decreases IEB permeability. S-layer induces an increased expression of the tight junction (TJ) transmembrane protein claudin-1, a structural TJ rearrangement and desmosomes’ formation. It also counteracts the reduction in alkaline phosphatase detoxification activity and the enhancement of pro-inflammatory interleukin-8 release both induced by LPS [191].

By targeting harmful bacteria, bacteriocins from certain postbiotics can indirectly support intestinal barrier function by reducing the presence of pathogens that may compromise the integrity of the IEB. Bacteriocins are ribosomally-synthesized secreted antimicrobial peptides capable of inhibiting both food spoilage/pathogenic bacteria from both Gram-negative and Gram-positive group. Class I bacteriocin nisin from *Lactococcus lactis* is known to inhibit the germination of *Clostridium botulinum* spores, while class II bacteriocin pediocin PA-1 from *Pediococcus acidilactici* inhibits the growth of *Listeria monocytogenes* [149,172].

## 6. Limitations

This review has several limitations that should be acknowledged. Firstly, although extensive, the selection of studies included was not systematic and may not encompass all relevant literature on the subject, potentially introducing selection bias. Secondly, the heterogeneity of study designs, populations, and interventions across the referenced literature complicates direct comparisons and limits the generalizability of findings. Thirdly, while the mechanistic insights into biotics’ effects are discussed, many of these mechanisms are derived from in vitro or animal studies, which may not fully translate to human contexts. Additionally, the rapid evolution of microbiome research and the emergence of novel biotic formulations mean that some findings may quickly become outdated. Finally, limitations in long-term clinical data hinder the ability to make conclusive statements regarding the safety, efficacy, and optimal application of prebiotics, probiotics, synbiotics, and postbiotics in various patient populations.

## 7. Conclusions

Various in vitro and in vivo studies have shed light on how biotics exhibit various bioactivities, such as modifying the microbiota in the gastrointestinal tract and improving the functioning of the intestinal epithelial barrier. However, the signaling pathways that underlie these actions still need to be entirely understood and require further investigation [149]. Current innovations in biotics formulations also focus on integrating genomics and biotechnological advancements. Understanding complex interactions and mechanisms is essential for developing more targeted and effective therapeutic strategies [227]. Furthermore, using adequate new technologies to identify their bioactive components is crucial to ensure product quality.

Advances in microbiome sequencing and analysis are paving the way for personalized nutrition strategies that tailor prebiotic and probiotic interventions to an individual’s unique microbiome composition. This personalized approach has the potential to maximize health benefits and minimize adverse effects.

The use of certain biotics is supported by thorough efficacy evaluations, yet not all products have undergone validation. The aim is to ensure healthcare professionals utilize these interventions based on scientific evidence.

## Figures and Tables

**Table 1:** Comparative overview of the source, function, stability, and safety of biotics. FOSs, fructooligosaccharides; GOSs, galactooligosaccharides; SCFAs, short-chain fatty acids.

Category	Source	Function	Stability	Safety
**Prebiotics**	Non-digestible dietary fibers (e.g., inulin, FOSs, GOSs)	Stimulate growth/activity of beneficial gut bacteria; SCFA production	High thermal and shelf stability	Generally recognized as safe and well tolerated with minimal side effects
**Probiotics**	Live microorganisms (e.g., *Lactobacillus*, *Bifidobacterium*, *Saccharomyces*)	Modulate gut microbiota; enhance barrier function and immunity; antimicrobial production	Sensitive to heat, pH, oxygen; viability must be preserved	Safety well-studied in healthy populations; caution in immunocompromised
**Synbiotics**	Combination of live microbes and substrate (e.g., *B. lactis* + inulin)	Enhance probiotic survival and activity; improved microbial balance and function	Stability depends on formulation; better in encapsulated forms	Safety similar to probiotics; combination must be assessed for interactions
**Postbiotics**	Inactivated microbial cells and/or their metabolites	Modulate immunity; reinforce barrier integrity; deliver microbial benefits without live organisms	Very stable under heat and storage; do not require cold chain	High safety profile; no risk of translocation or infection

**Table 2:** Summary of the major findings on various prebiotics.

Prebiotic Type	Sources	Effects	Ref.
Polydextrose (PDX)	Polysaccharide, randomly bonded glucose polymers	Modulates gut microbiota, reduces inflammation	[8,30]
Dextrins	Hydrolized starch and glycogen	Enhances short-chain fatty acid (SCFA) production, increases satiety, promotes beneficial gut bacteria, decreases *Clostridium* spp., reduces β-glucosidase and β-glucuronidase activities	[4,9,31,32]
Inulin (oligofructose-enriched inulin)	Chicory root, garlic, leeks, artichokes	Increases *Bifidobacterium*, improves bowel movements, improves lipid metabolism antioxidant and anti-inflammatory	[33,34,35]
Soluble corn fiber	Corn	Improves digestive health, alters gut microbiota composition	[36]
Resistant starch type 4	Chemically modified starches	Potential use in metabolic health management, increases resistance to enzymatic digestion	[37]
Fructooligosaccharide (FOS)	Chicory roots, onions, garlic, asparagus	Promotes growth of *Bifidobacterium* and *Lactobacillus* spp., increases colonic crypt size	[38,39,40]
Galactooligosaccharide (GOS)	Human milk, soybeans	Enhances immune function, reduces pathogen colonization	[41,42]
Arabinoxylan-oligosaccharides	Cereal grains	Improves gut health	[43]
Resistant starch type 1	Grains, seeds, legumes, pastas	Improves insulin sensitivity, physically inaccessible starch; passes through the small intestine undigested	[44,45,46,47,48]
Resistant starch type 2	Green bananas, raw potatoes, high amylose corn, specific legumes	Increases production of SCFAs, contains raw starch granules; resistant to enzymatic digestion	
Resistant starch type 3	Cooked and cooled starchy foods like bread, cakes, cornflakes	Enhances satiety, reduces fat storage	
Resistant starch type 4	Chemically modified starches	Potential use in metabolic health management, increases resistance to enzymatic digestion	
Resistant starch type 5	Starch–lipid complexes formed during food processing	Resistant to amylolytic hydrolysis; improves gut health by passing undigested	
Wheat, oat, corn, barley, rye bran	Cereal brans, whole grains	Enhances stool bulk, supports gut microbiota diversity	[49,50,51]
Fruit/vegetable fibre	e.g., lupin kernel, sugar cane, bean, citrus, various fruit	Supports gut microbiota	[52,53]

**Table 3:** Summary of the major findings on various probiotic species and their effects on the gut. IBD = inflammatory bowel diseases; IL = interleukin; NF-κB = nuclear factor kappa light-chain enhancer of activated B cells; TNF-α = tumor necrosis factor α.

Probiotic Species	Major Findings	Ref.
*Lactobacillus acidophilus*	Exhibits anti-inflammatory effects, enhances IL-17 and IL-22 production, improves colitis symptoms when used with Veillonella ratti, and upregulates protective cytokines	[58,59]
*Lactobacillus fermentum*	Reduces chronic gut inflammation by increasing IL-6 and IL-10, inhibits harmful bacteria, protects against gut permeability from chemotherapy, and reduces inflammation through the NF-κB pathway	[60,61,62]
*Lacticaseibacillus rhamnosus*	Lowers IL-18 levels, boosts IL-10, helps recover body weight and colon length in colitis, improves disease markers, strengthens the epithelial barrier, and promotes regeneration of intestinal stem cells	[63,64]
*Ligilactobacillus salivarius*	Reduces pro-inflammatory markers, enhances epithelial barrier function, and prevents intestinal pathogens from adhering	[65,66]
*Limosilactobacillus mucosae*	Protects against experimental colitis by upregulating colonic 5-HT4 and TGF-β2, as well as alleviates colitis symptoms	[67]
*Bifidobacterium longum*	Promotes healing of wounds, lowers IL-6 and TNF-α levels, improves colitis, enhances immune response when combined with B. bifidum, and fortifies the epithelial barrier	[68,69]
*Bifidobacterium breve*	Reduces colitis symptoms, increases goblet cell count, strengthens epithelial barrier, and decreases oxidative stress	[70,71]
*Bifidobacterium bifidum*	Ameliorates colitis symptoms, restores body weight and colon length, strengthens epithelial barrier, and increases anti-inflammatory factors; protective against non-alcoholic fatty liver disease	[72,73]
*Saccharomyces cerevisiae (yeast)*	Reduces TNF-α, increases IL-10, protects against colitis, and suppresses macrophages pyroptosis	[74,75,76]
*Faecalibacterium prausnitzii*	Decreases disease scores and significantly reduces inflammation in IBD	[77]
*Enterococcus faecium*	Enhances epithelial barrier, lowers pro-inflammatory cytokines, reduces inflammation in obesity, and reduces inflammation through the NF-κB pathway	[78,79]

**Table 4:** Overview of selected clinical studies assessing the impact of prebiotics, probiotics, synbiotics, and postbiotics on intestinal barrier function. AAD, antibiotic-associated diarrhea; FOSs, fructooligosaccharides; NEC, necrotizing enterocolitis.

Study Population	Intervention	Outcome	Clinical Relevance	Ref.
Preterm infants (NEC)	Probiotics (*Lactobacillus*, *Bifidobacteria*)	Improved epithelial barrier, reduced inflammation	Supports prevention of NEC in neonates	[92,93,94]
Obese individuals	Prebiotics	Improved gut barrier, reduced inflammation, enhanced insulin sensitivity	Potential therapeutic use for obesity and metabolic disorders	[95,96,97]
Adults with infectious diarrhea	Probiotics (*S. boulardii*, *L. reuteri*, *B. lactis*)	Reduced duration and incidence of diarrhea	Effective for traveler’s and acute diarrhea treatment	[98,100]
Patients with antibiotic-associated diarrhea (AAD)	Probiotics (*S. boulardii*, *L. rhamnosus* GG, multi-strain)	Reduced AAD occurrence, restored gut microbiota	Prevents common AAD complications	[102,104]
Cancer patients (chemo-/radiation-induced diarrhea)	Probiotics (*L. acidophilus*, *B. bifidum*, *Saccharomyces*)	Reduced diarrhea severity and frequency	Supports gut integrity during cancer treatment	[107,108]
Children with functional diarrhea	Prebiotics (FOS)	Improved microbiota balance, symptom relief	Alternative to antibiotics in pediatric diarrhea	[109]
Adults with metabolic syndrome	Synbiotic supplementation	Improved gut microbiota composition; reduced inflammation markers	Potential adjunct therapy for metabolic disorders	[2]
Patients undergoing stem cell transplantation	Probiotic and prebiotic administration	Modulated gut microbiota; reduced transplant-related complications	Supportive care during transplantation	[154]
Individuals with skin aging concerns	Probiotic and prebiotic supplementation	Improved skin health; gut–skin axis enhancement	Dermatological benefits of gut modulation	[155]
Cancer patients undergoing chemotherapy	Postbiotic supplementation	Reduced gastrointestinal side effects	Improved tolerability of chemotherapy	[156]
Patients with Parkinson’s disease	Probiotic and prebiotic interventions	Alleviated GI symptoms; possible neurological improvement	Supportive therapy in neurodegenerative disease	[157]

## Abbreviations

AAD	antibiotic-associated diarrhea
BB-12	bifidobacterium animalis subsp. lactis strain bb-12
EPS	exopolysaccharide
FDA	food and drug administration
FFAR2/3	free fatty acid receptor 2/3
FFAs	free fatty acids
FOSs	fructooligosaccharides
GALT	gut-associated lymphoid tissue
GLP-1	glucagon-like peptide-1
GOSs	Galactooligosaccharides
GPCRs	g-protein coupled receptors
GPR109A	hydroxycarboxylic acid receptor hcar2
GPR41/43	g protein-coupled receptor 41/43
HDACs	histone deacetylases
HIF	hypoxia-inducible factor
IBD	inflammatory bowel disease
ICs	intestinal cells
IEB	intestinal epithelial barier
IL	Interleukin
ILC3	innate lymphoid cells type 3
INF	intestinal inflammation
ISAPPs	international scientific association for probiotics and prebiotics
ITFs	inulin-type fructans
LTA	lipoteichoic acid
MAPKs	mitogen-activated protein kinases
MUC2	Mucin-2
NF-κB	nuclear factor kappa-light-chain-enhancer of activated b cells
NGPs	next-generation probiotics
PDX	Polydextrose
PP	plasma protein
PS	Postbiotics
PYY	peptide YY
SCFAs	short-chain fatty acids
SIBO	small intestinal bacterial overgrowth
TJ	tight junction
TNF-α	tumor necrosis factor alpha
ZO-1	zonula occludens-1
